# Reduction of *Escherichia coli* O157:H7, *Listeria* *monocytogenes*, and Naturally Present Microbe Counts on Lettuce using an Acid Mixture of Acetic and Lactic Acid

**DOI:** 10.3390/microorganisms7100373

**Published:** 2019-09-20

**Authors:** Jiayi Wang, Yeting Sun, Dongbing Tao, Shan Wang, Chen Li, Fenge Zheng, Zhaoxia Wu

**Affiliations:** 1 College of Food Science, Shenyang Agricultural University, 120 Dongling Rd., Shenyang 110866, China; jiayiwangsyau@syau.edu.cn (J.W.); syt_nercv@163.com (Y.S.); dbtt@163.com (D.T.); wangshan@syau.edu.cn (S.W.); lichen940329@163.com (C.L.); 2 Vegetable Research Center, Academy of Agriculture and Forestry Sciences, Beijing 100097, China; 3 Shenyang Product Quality Supervision and Inspection Institute, Glide Rd, Shenyang 110136, China; Syzfe2012@163.com

**Keywords:** organic acid, acid mixture, disinfection, 16S rRNA, minimal processing

## Abstract

Lactic acid (LA) and acetic acid (AA) are independently used to disinfect fresh leaf vegetables. LA has a higher efficacy but costs more than AA. Herein, we compared the disinfection efficacy of LA, AA, and their mixture on lettuce to determine whether the cheaper acid mixture shows similar or more efficacy than LA. Quality analysis indicated that the acid mixture and individual acids did not cause additional loss of instrument color and polyphenolic content compared with that of the control; however, visible defects were observed at AA concentrations exceeding 0.8%. Analysis of *Escherichia coli* O157:H7, *Listeria* *monocytogenes*, and naturally present microbes (aerobic mesophilic and psychrotrophic bacteria, coliforms, molds, and yeasts) showed that the acid mixture led to the highest reduction in microbial count during storage. 16S rRNA sequencing was further employed to understand the effects of the acid mixture and individual acids on lettuce microbial ecology. During storage, the acid mixture and individual acids significantly decreased the abundance of *Massilia* spp. and *Alkanindiges* spp. but there was a marked increase in *Escherichia-Shigella* abundance (LA: 0.003–58.82%; AA: 0.01–55.34%; acid mixture: undetected to 50.71%; control: 0.007–33.09%), indicating that acid disinfection altered the microbial ecology to stimulate *Escherichia-Shigella* growth. These results enhance our understanding of the relationship between lettuce disinfection and ecological changes.

## 1. Introduction

Consumption sanitizers are classified into two types—organic acids and oxidizing agents [1]. Most organic acids have been approved as generally recognized as safe (GRAS) compounds by the Food and Drug Administration and are used as pH regulators and preservatives in the food industry. Their antibacterial mechanisms are mainly based on environmental pH reduction, cellular anion accumulation, and cellular pH reduction [2]. Among them, lactic acid (LA) and acetic acid (AA) are commonly used in minimal processing [3]. The disinfection effects of LA and AA on produce have been reported, and LA has been shown to be a better antimicrobial agent in reducing aerobic mesophilic counts (AMC), *Listeria monocytogenes*, and *Escherichia coli* O157:H7 as compared to AA [1,4,5,6].

The combined use of an organic acid and oxidizing agent is more effective than the use of one sanitizer alone, as the dissociated acid molecules and oxidizing agent can cause protein agglutination and membrane destruction, respectively. Organic acids and aqueous ozone were reported to exhibit enhanced efficacy in disinfection against *E. coli* O157:H7, *Shigella* spp., and *L. monocytogenes* [7,8,9]. Additional microbial reduction was also observed when using an organic acid and electrolyzed water as well as organic acid and hydrogen peroxide for lettuce disinfection [10]. The combination of acids also achieved more effective disinfection results. The synergistic effect of LA and AA has been observed in culture medium [11] and an acid mixture of LA and AA is used as a preservative in fermented foods [12,13] and to disinfect meat products [14]. However, the surface structure of fresh leaf vegetables differs from that of meat products; for instance, lettuce has stomata and leaf veins as well as a curved structure that may provide an ideal environment for bacteria [3]. Meat, on the other hand, has a porous structure with high protein and fat contents that can act as adhesive particles. In addition, the bacterial composition differs between meat and leaf vegetables. Thus, whether an acid mixture can lead to additional reduction in microbial counts on fresh produce remains unclear. Moreover, based on microbial count analysis, differences in the microbial reduction between the acid mixture and individual acids led to the question of whether these treatments exert different effects on the micro-ecology of lettuce. Therefore, the objectives of this study were as follows: (1) to compare the effects of an acid mixture on lettuce microbial counts (*E. coli* O157:H7, *Listeria monocytogenes*, and those naturally present), color, and polyphenolic content during storage; and (2) to understand the effects of the acids on lettuce surface ecology during storage via culture-independent 16S rRNA sequencing.

## 2. Materials and Methods

### 2.1. Sample Preparation

Green leaf lettuce (*Lactuca sativa* L. var. *crispa*; 200–220 g; without mechanical damage), which has a thinner and more fragile leaf structure than iceberg lettuce, was selected as the experimental sample. Lettuce was purchased from a local market (Maguanqiao street, Shenhe district, Shenyang, China) on the day of the experiment. After removing the two outer leaves, inner baby leaves, and stem, the samples were rinsed with tap water to remove dirt and then cut into pieces using a circular cutting edge (diameter of 5.2 × 10^−2^ m) [1]. The cut samples were dewatered for 30 s using an ethanol-sterilized manual salad spinner.

### 2.2. Inoculation of Lettuce Leaves

Non-toxic *E. coli* O157:H7 (NCTC12900) and *L. monocytogenes* (ATCC19115) were kindly provided by Dr. Yeting Sun from the Vegetable Research Center, Beijing Academy of Agriculture and Forestry Sciences. The obtained culture was stored in glycerol solution at a ratio of 1:1. Before use, *E. coli* O157: H7 and *L. monocytogenes* were purified using sorbitol MacConkey agar (SMAC; Hopebio, Qingdao, China) and *Listeria* chromogenic agar (Land Bridge, Beijing, China). Next, single colonies were inoculated into tryptic soy broth medium (TSB; Hopebio) to prepare the working culture. The inoculation procedure was previously described by Huang and Chen [5] and was used with slight modifications. Briefly, the working culture count was adjusted to approximately 10^9^ CFU/mL at an OD_600_ = 0.25 for *E. coli* O157: H7 and an OD_600_ = 0.32 for *L. monocytogenes*. Next, 5 mL of the adjusted cell suspension was mixed with 200 mL of 8.5 g/L NaCl in a sterilized plastic bag, after which 10 g of the lettuce leaves was submerged in the cell suspension and gently massaged for 5 min. Cell suspensions were drained and the lettuce leaves were placed on a sterilized gauze and dried in a biosafety hood at room temperature (25 ± 1 °C) for 30 min. Finally, the leaves were placed in a sterilized plastic box for 24 h at 4 °C to facilitate bacterial attachment.

### 2.3. Disinfection

LA (90% purity; Macklin, Shanghai, China), AA (analytical grade; Macklin), and their mixture were prepared at the indicated concentrations (0.8% LA + 0.2% AA, 0.6% LA + 0.4% AA, 0.4% LA + 0.6% AA, 0.2% LA + 0.8% AA, 1% LA, and 1% AA). The lettuce sample (40 g) was placed in an 800 mL acid solution and shaken for 1.5 min under 150 rpm [2]. The disinfected sample was then transferred into tap water for 15 s to remove the acid residue. After removing excess water from the lettuce surface using a salad spinner, 30 g of the sample was transferred to a polyethylene terephthalate box (18 × 13 × 4 cm), which was then sealed using a polyvinyl chloride cling film (Nan Ya, Tai Wan, China). Owing to the short shelf life of minimally processed products and consumer demand for fresh products, the sample was stored for 5 days at 5 °C. Each treatment was performed independently three times. A non-disinfected sample was used as a control.

### 2.4. Microbiological Analysis

*E. coli* O157:H7, AMC, aerobic psychrotrophic count (APC), coliforms, and molds and yeasts (M&Y) were analyzed immediately after acid disinfection and at the end of the storage period (day 5). At the sampling points, 10 g was sampled and each piece was divided into four parts to ensure full contact with the NaCl solution. Next, 5 g of the sample was placed in an Erlenmeyer flask containing 70 mL of 8.5 g/L NaCl solution and shaken for 3 min at 260 rpm to prepare a 15-fold dilution, after which 6 mL of the suspension was added to 34 mL of 8.5 g/L NaCl solution to prepare a 100-fold dilution [2]. Other dilutions were prepared as needed. A 1 mL dilution was pour-plated onto (1) plate count agar (Hopebio) and incubated at 37 °C for 2 days for the measurement of AMC, as well as at 7 °C for 10 days for the measurement of APC and (2) violet red bile glucose agar (VRBGA; Hopebio) and incubated at 37 °C for 1 day for the measurement of coliforms. Moreover, a 0.1 mL dilution was surface-plated on (3) rose Bengal agar (Hopebio) and incubated at 30 °C for 3 days for the measurement of M&Y; (4) SMAC agar (Hopebio) and incubated at 37 °C for 1 day for the measurement of *E. coli* O157:H7; and (5) *Listeria* chromogenic agar (Land Bridge) and incubated at 37 °C for 1 day for the measurement of *L. monocytogenes*. The results are expressed as microbial reductions (log CFU g^−1^).

### 2.5. Color and Polyphenolic Content Analysis

Instrument color and polyphenolic content were analyzed at the end of the storage period. For the instrument color, 10 pieces were selected and the L^*^, a^*^, and b^*^ values for two locations per piece were read using a colorimeter (CR400; Konica Minolta, Osaka, Japan). Among the L*, a*, and b* parameters, negative to positive values represented darkness to light, green to red, and blue to yellow, respectively. The colorimeter was calibrated using a white standard plate (*Y* = 82.80, *x* = 0.3194, *y* = 0.3264) before each use.

For polyphenolic content analysis, 5 g of fresh sample was extracted using 75 mL of 80% methanol in a blender for 2 min. After allowing the sample to stand for 2 h at 4 °C, the slurry was filtered and centrifuged at 12,000× *g* for 10 min [2]. Polyphenolic content was determined according to the Folin-Ciocalteu method [15] with some modifications. Briefly, 50 µL of the suspension was added to 3 mL of distilled water and oxidized with 250 μL of Folin reagent. After allowing to stand for 6 min, the reaction was neutralized using 750 μL of 200 g/L sodium carbonate and then incubated for 90 min in the dark. The absorbance was read at 765 nm and calculated according to a standard curve (*y* = 0.0015*x* + 0.0006, *R*^2^ = 0.9981; the *x*-axis indicates mg L^−1^ gallic acid, the *y*-axis indicates the absorbance at 765 nm). The results are expressed as gallic acid equivalents (GAE) in mg kg^−1^ (expressed on a fresh weight basis).

### 2.6. DNA Extraction and Microbial Verification

After analyzing the microbial count data, an effective acid mixture (i.e., 0.8% AA + 0.2% LA), 1% AA, 1% LA, and the control were selected, after which 16S rRNA sequencing was performed to detect naturally present microbes. The microbial count was analyzed once more to ensure that the results obtained showed a similar trend with Section 2.4. The disinfection process was performed as described in Section 2.3. The microbial suspension was prepared as described in Section 2.4. For total microbial DNA extraction, 40 mL of the suspension was drawn with a sterile syringe and filtered through two Millipore membranes (0.22 μm; 20 mL each; Billerica, MA, USA), after which DNA was extracted from the resulting membranes using Fast DNA SPIN extraction kits (MP Biomedicals, Santa Ana, CA, USA) according to the manufacturer’s instructions. DNA quality and concentration were determined by agarose gel electrophoresis and a NanoDrop ND-1000 spectrophotometer (Thermo Fisher Scientific, Waltham, MA, USA), respectively. The agar type and culture conditions are described in Section 2.4. Each treatment was replicated six times. Verification of microbiological analysis was performed three times with six replicates, and the results are expressed as microbial reduction (log CFU g^−1^).

### 2.7. Amplicon Pyrosequencing

The bacterial 16S rRNA V3-V4 region was amplified by PCR with each sample containing 5 μL of Q5 reaction buffer (5×), 5 μL of Q5 High-Fidelity GC buffer (5×), 0.25 μL of Q5 High-Fidelity DNA Polymerase (5 U μL^−1^), 2 μL of 2.5 mM dNTPs, 1 μL (10 µM) of each forward (338F; 5′-ACTCCTACGGGAGGCAGCA-3′) and reverse (806R; 5′-GGACTACHVGGGTWTCTAAT-3′) primer, 2 μL of template DNA, and 8.75 μL of ddH_2_O. PCR conditions were as follows: initial denaturation at 98 °C for 2 min followed by 25 cycles of denaturation at 98 °C for 15 s, annealing at 55 °C for 30 s, extension at 72 °C for 30 s, and a final extension at 72 °C for 5 min. The resulting PCR amplicons were purified using Agencourt AMPure Beads (Beckman Coulter, Brea, CA, USA) and quantified using the PicoGreendsDNA Assay Kit (Invitrogen, Carlsbad, CA, USA). After pooling into equal amounts, the obtained amplicons were sequenced using the Illumina MiSeq platform with MiSeq Reagent Kit v3 (San Diego, CA, USA) at Shanghai Personal Biotechnology Co., Ltd. (Shanghai, China).

### 2.8. Sequence Processing and Bioinformatics Analysis

The paired-end reads were conducted using FLASH [16]. The quality of the sequences was controlled by screening the data using Quantitative Insights Into Microbial Ecology (QIIME, v1.8.0) software as described by Caporaso et al. [17]. The raw sequencing reads with exact matches to the barcodes were classified for each sample and identified as valid sequences. Reads with sequence lengths < 150 bp, average Phred scores < 20, and containing ambiguous bases and mononucleotide repeats >8 bp were excluded as low-quality data [18]. After chimera detection, sequences with 97% similarity were classified into operational taxonomic units (OTUs) using UCLUST [19]. Next, default parameters were used to select a representative sequence, which was imported into the BLAST system to classify the OTUs against the Greengenes Database using the best hit. The resulting OTUs containing less than 0.0001% of the total sequences (all samples) were discarded. An OTU table was created after averaging the 100 OTU subsets that were resampled under the 90% minimum sequencing depth. Based on this table, biodiversity estimators (i.e., ACE, Chao 1, Shannon, and Simpson indices) were calculated using QIIME.

### 2.9. Statistical Analysis

The data were compared by one-way analysis of variance using SPSS v.20 and the differences in means were analyzed by Duncan’s multiple range tests. Significant differences between day 0 and day 5 were analyzed by independent samples *t* test. *p* < 0.05 was considered statistically significant.

## 3. Results and Discussion

### 3.1. Effects of the Acid Mixture on Quality Properties

**Figure 1:**
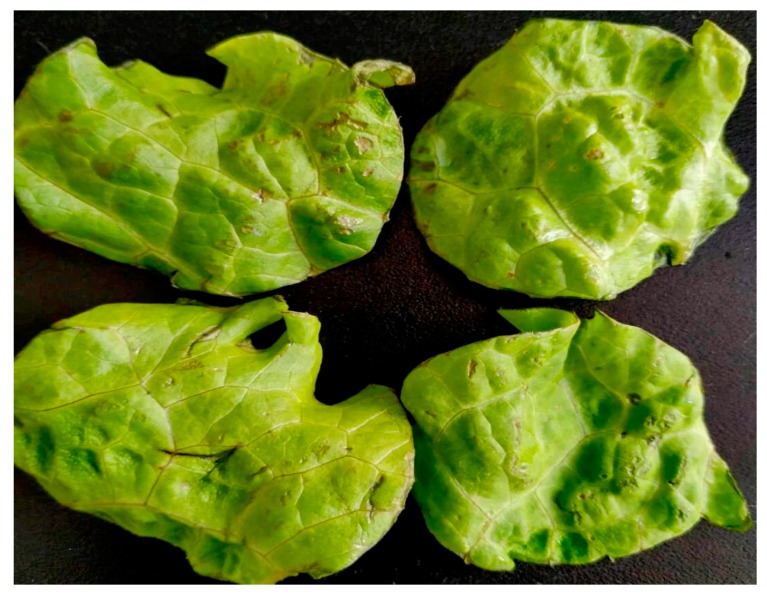
Visual quality-loss of lettuce samples after washing with 1% acetic acid and storage for 5 days.

After storage for 5 days, the L^*^, a^*^, and b^*^ values in the different treatment groups were similar to those in the control group and were not significantly different from each other (Table 1). However, when the AA concentration increased to 1%, visible defects were observed on lettuce leaves (Figure 1). Similarly, Van Haute et al. [20] concluded that the quality of sugar snaps is reduced once the AA concentration exceeds 0.8%. Polyphenolic content analysis showed similar results between the acid treatment groups and control group, ranging from 272.95 to 291.27 mg kg^−1^ GAE (Table 1). These results indicate that disinfection with 1% LA and the acid mixture does not lead to additional changes in color and phenolic loss compared with that of the control treatment and tap water only treatment.

### 3.2. Effects of Acid Mixture on Microbial Counts

**Figure 2:**
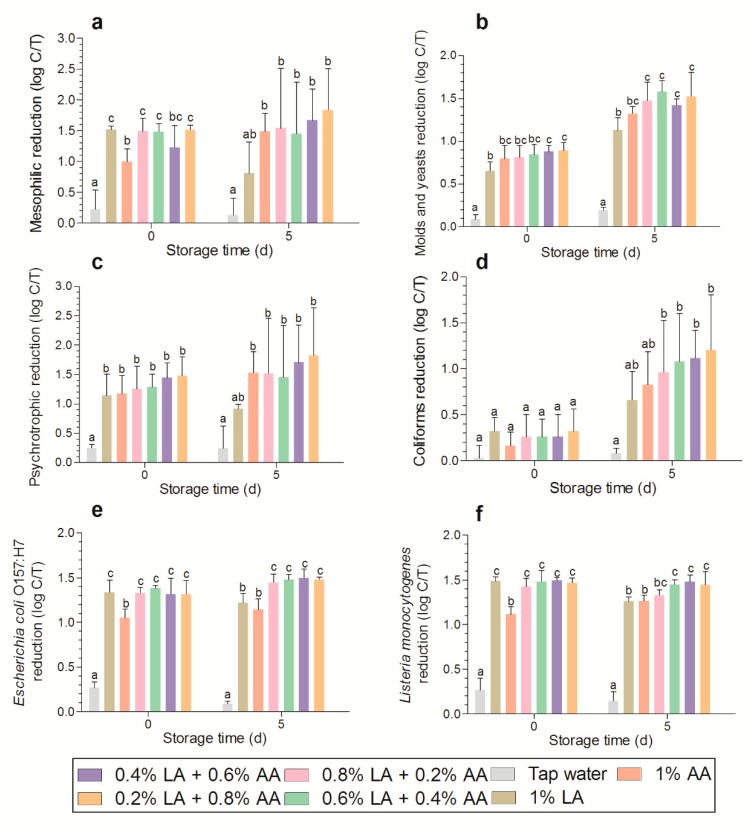
Microbial reduction of aerobic mesophilic counts (**a**), molds and yeasts (**b**), aerobic psychrotrophic counts (**c**), coliforms (**d**), inoculated *E. coli* O157:H7 (**e**), and inoculated *L. monocytogenes* (**f**) present on lettuce. Data represent the means ± standard deviation, and the different letters above the columns indicate significant differences (*p* < 0.05). LA, lactic acid; AA, acetic acid. Log C/T indicates the difference in microbial counts between the control and treatment groups at the same time points.

The aerobic mesophilic counts of the control group were 5.43 ± 0.62 log CFU g^−1^ at day 0 and 7.13 ± 0.40 log CFU g^−1^ at day 5; aerobic psychrotrophic counts were 5.22 ± 0.74 log CFU g^−1^ at day 0 and 7.24 ± 0.49 log CFU g^−1^ at day 5; M&Y counts were 3.95 ± 0.26 log CFU g^−1^ at day 0 and 5.87 ± 0.15 log CFU g^−1^ at day 5; coliform counts were 2.52 ± 0.07 log CFU g^−1^ at day 0 and 5.65 ± 0.41 log CFU g^−1^ at day 5; *E. coli* O157:H7 counts were 6.76 ± 0.15 log CFU g^−1^ at day 0 and 7.82 ± 0.06 log CFU g^−1^ at day 5; *L. monocytogenes* counts were 6.59 ± 0.21 log CFU g^−1^ at day 0 and 7.27 ± 0.17 log CFU g^−1^ at day 5. After acid disinfection, the acid mixture showed similar effects to those of LA and better effects than those of AA in the reduction of AMC, *L. monocytogenes*, and *E. coli* O157:H7 counts (*p* < 0.05) (Figure 2). After storage for 5 days, a non-significant difference was observed between LA and AA, while the acid mixture showed better effects than those of LA (1.45–1.50 log *E. coli* O157:H7 reduction for acid mixture vs. 1.22 log for LA). This may be associated with changes in the ecological environment after disinfection. Similarly, Poimenidou et al. [21] found that *E. coli* O157:H7 was undetectable on stored lettuce washed with vinegar; however, the count in the sample treated with LA was approximately 3 log. Another study showed that 1% organic acids significantly reduced the count growth of *L. monocytogenes*, whereas 0.5% organic acids stimulated its growth; the authors suggested that this may be because *L. monocytogenes* is more resistant to 0.5% organic acids and is more competitive than the native microflora [6]. In the present study, after storage for 5 days, *L. monocytogenes* counts were significantly reduced in the acid mixture group (except for 0.2% AA + 0.8% LA) compared with the 1% LA and 1% AA groups (Figure 2f).

According to some studies, the trend of APC is similar to that of AMC [10,22]. In this study, a non-significant difference between AA and LA was observed for APC reduction at day 0 (Figure 2c), which was inconsistent with the trend observed for AMC. Similarly, the reduction in the M&Y microbial count by AA was higher than that by LA (Figure 2b); this may be due to the presence of nuclear membranes in fungi as dissociated acetate anions can more easily penetrate nuclear membranes to exert toxic effects on DNA as compared to lactate anions. Compared with 1% LA, 0.2% LA + 0.8% AA at day 0 showed a significantly increased reduction in microbial count (0.65–0.89 log). After storage for 5 days, the reduction in M&Y in the acid mixture group was significantly higher than that in the LA group. The reduction in coliform count was not more than 0.5 log (Figure 2d), which is consistent with the findings of Martínez-Sánchez et al. [22]. However, during subsequent storage, microbial count reductions by AA and LA were 0.83 and 0.66 log, respectively, which were not significantly higher than those observed in the tap water group. On the other hand, the microbial count reduction by the acid mixture was significantly higher than that achieved by tap water, with a reduction ranging from 0.96 to 1.21 log. In summary, the acid mixture led to the highest microbial reduction during storage. Moreover, the cost of AA is generally lower than that of LA; thus, the acid mixture should be considered as an alternative to traditional treatment with individual acids.

### 3.3. Effects of Acid Mixture on Biodiversity

At the postharvest stage, 16S rRNA sequencing has been successfully used to analyze changes in bacterial and fungal communities during refrigeration. Lopez-Velasco et al. [23] showed that refrigeration can decrease the richness and evenness of the microbial community on spinach, particularly after storage for 15 d. Interestingly, Shen et al. [24] reported that refrigeration can increase the richness and evenness of fungal communities on apples. They also suggested that the long-term storage of apples is associated with a higher risk of mycotoxin contamination by molds belonging to the genera *Penicillium*, *Aspergillus*, *Mucor*, and *Botrytis*. However, the changes that occur in the bacterial community after disinfection and during refrigeration are unclear. In this study, according to the microbial verification results, we examined whether the microbial reduction trend observed in this experiment was similar to that observed in Figure 2 and confirmed it to be true. ACE and Chao 1 indices indicate the extent of microbial richness and, after storage for 5 days, these indices were not significantly decreased in the control group (Table 2); however, the indices of the disinfection groups were significantly decreased and similar to each other. Similarly, when accounting for the extent of microbial evenness, the Simpson and Shannon index values of the acid disinfection groups were significantly decreased after storage and those of the acid mixture group were similar to those of the 1% LA and 1% AA groups (Table 2). These results indicate that acid disinfection can significantly reduce biodiversity during storage and that the effects of the acid mixture on biodiversity were similar to those of individual acids.

### 3.4. Effects of Organic Acid and Acid Mixture on Bacterial Composition

At the genus level, *Pseudomonas*, *Alkanindiges*, *Massilia*, *Sphingomonas*, and *Acinetobacter* were the dominant genera (Table 3). Of these, *Pseudomonas* spp. are responsible for food spoilage [25] and frost damage [26]. Interestingly, *Pseudomonas* spp., as biocontrol organisms (e.g., *Pseudomonas fluorescens* WCS365) [27], can function by inducing systemic resistance [28] and cause a dramatic decrease in succinic and citric acids. For example, in the rhizosphere, *Pseudomonas* spp. can be attracted by malic, citric [29], and fusaric acids [30]. In this study, disinfection with the acid mixture led to the highest abundance of *Pseudomonas*, which was higher than that of the control samples and significantly higher than that of the AA- and LA-treated samples. We hypothesize this may be because, compared with other genera, *Alkanindiges* and *Sphingomonas* are more resistant to AA and *Acinetobacter* is more resistant to LA. When using the acid mixture, the disinfection efficacy was improved. The extent of improvement for *Pseudomonas* was lower than that for the other genera; thus, the abundance of *Pseudomonas* in the acid mixture group was higher than that in the AA and LA groups, and even higher than that in the control group. The antibacterial mode of action of AA is similar to that of LA, i.e., undissociated acid molecules attach to the bacterial membrane and, after penetration, the higher cellular pH environment stimulates the acid mixture to dissociate into lactate and acetate anions; these anions exhibit their toxic effects by acting on DNA, RNA, and ATP [31]. However, Ricke [32] suggested that the relationship between energy dissipation and ATP production is complex and proposed that acid-sensitive protein denaturation is the major antibacterial mode of action. These insights are consistent with the results of Wang et al. [33]. Based on this, we further hypothesize that because of the different cellular protein structures and extracellular polysaccharides of different bacteria, the binding sites of lactate and acetate anions are also different, making some specific bacteria resistant to disinfection and leading to different bacterial communities after disinfection. Additionally, through the combined action of lactate and acetate anions, the disinfection efficacy may be improved by affecting multiple protein sites. This hypothesis should be verified in further studies using proteomics and cryo-electron microscopy or nuclear magnetic resonance analysis. Overall, these results indicate that the acid mixture and individual acids have different effects on lettuce microbial ecology.

*Pantoea*, *Pseudomonas*, *Klebsiella*, *Enterobacter*, *Aeromonas*, and *Burkholderia* inhibit *E. coli* O157:H7 [34], which may explain the unchanged abundance of *Pseudomonas* after storage. *Massilia* is positively associated with the root microbiome succession at an early stage during plant growth and has the potential to control plant pathogens such as *Pythium aphanidermatum* [35]. Unexpectedly, the abundance of *Massilia* after storage in the acid disinfection groups was significantly lower than that in the control group. *Alkanindiges* abundance is an indicator of lettuce health [36]; its abundance was not affected after acid disinfection, but was significantly decreased during storage and significantly lower than that of the control group at day 5, indicating that acid disinfection may affect lettuce ecology and promote the interaction of other microbes with *Alkanindiges*.

Among the four groups, the LA treatment group led to the highest *Escherichia-Shigella* abundance, which was significantly higher than that of control. The next highest abundance values were found in the AA and acid mixture groups. These results indicate that acid disinfection can stimulate Enterobacteriaceae growth. When considering the coliforms and *E. coli* O157:H7, microbial counts were not higher than that of the control group at day 5. If the storage time is prolonged, *Escherichia-Shigella* may show its co-exclusion activity against other microbes, and the counts may continue to grow to values even higher than that of control. Also taking into consideration the visible defects caused by 1% AA at day 5, the relationship between ecology changes, storage day, quality, and microbial reduction should be integrated to evaluate an efficient disinfection method. It has been reported that the plated counts of the sanitizer-treated group were similar or higher than those of the control group during storage, irrespective of the initial aerobic plate counts [20,22,37,38]. Our study was performed from an ecological perspective to explain this previously observed phenomenon. For the control group, the increased abundance of *Escherichia-Shigella* suggests co-exclusion activity against *Sphingomonas* and *Acinetobacter*, which has not been reported in microbial interaction databases.

## 4. Conclusions

This study provides evidence that an acid mixture can serve as an alternative to individual acids for disinfecting fresh produce. However, the individual acids and acid mixture altered the ecological environment, stimulating *Escherichia-Shigella* growth. Considering the different modes of action, a combination of acid mixtures and oxidizing sanitizers may be a promising approach to controlling the abundance of *Escherichia-Shigella*. Recently, minimizing wash water usage and preventing cross-contamination have received more attention, and thus many studies have demonstrated the cost-effective quality and the cross-contamination-prevention abilities of chlorine-based sanitizers compared with sanitizers used independently. In subsequent studies, we will compare acid mixtures and chlorine-based sanitizers to prevent wash water cross-contamination. Moreover, changes in the wash water bacterial community have not been widely studied; thus, we will use 16S rRNA sequencing to reveal the dominant bacterial taxa in wash water after washing. We then wish to utilize these findings as a basis for designing water disinfection and purification strategies to minimize wash water and sanitizer consumption and decrease cross-contamination risk.

## Figures and Tables

**Table 1:** Instrument color and total phenolic content of the disinfected sample stored for 5 days at 5 °C.

Treatment	Polyphenolic Content (mg kg^−1^ GAE)	Instrument Color		
		L *	-a *	B *
Control	299.69 ± 25.05 ^a^	51.74 ± 2.13 ^a^	18.70 ± 0.80 ^a^	34.37 ± 1.66 ^a^
Tap water	295.41 ± 19.16 ^a^	53.11 ± 1.27 ^a^	19.22 ± 0.82 ^a^	35.86 ± 1.55 ^a^
1% AA	291.27 ± 38.46 ^a^	50.68 ± 1.07 ^a^	19.60 ± 0.89 ^a^	36.31 ± 1.36 ^a^
1% LA	286.57 ± 40.10 ^a^	53.27 ± 0.70 ^a^	19.26 ± 0.82 ^a^	36.89 ± 1.86 ^a^
0.8% LA + 0.2% AA	266.63 ± 44.71 ^a^	52.19 ± 2.19 ^a^	19.40 ± 0.59 ^a^	35.82 ± 1.59 ^a^
0.6% LA + 0.4% AA	283.91 ± 42.06 ^a^	50.85 ± 0.70 ^a^	19.48 ± 0.53 ^a^	36.10 ± 0.64 ^a^
0.4% LA + 0.6% AA	272.95 ± 37.42 ^a^	50.65 ± 2.31 ^a^	19.22 ± 0.69 ^a^	35.29 ± 1.50 ^a^
0.2% LA + 0.8% AA	276.31 ± 19.13 ^a^	50.53 ± 3.84 ^a^	19.42 ± 1.02 ^a^	35.43 ± 3.07 ^a^

Values are expressed as mean ± standard deviation. Different letters in the same column indicate significant differences (*p* < 0.05). AA, acetic acid; LA, lactic acid; GAE, gallic acid equivalent.

**Table 2:** Biodiversity estimator and operational taxonomic unit (OTU) numbers after disinfection.

Parameter		Control	1% AA	1% LA	0.2% LA + 0.8% AA
Total OTU	day 0	451 ± 79	481 ± 57	487 ± 90	463 ± 62
	day 5	395 ± 36	333 ± 54	337 ± 38	340 ± 40
Genus OTU	day 0	425 ± 72	448 ± 59	460 ± 89	435 ± 62
	day 5	387 ± 33	330 ± 52	333 ± 37	336 ± 38
Chao1	day 0	532.69 ± 126.11 ^Aa^	550.94 ± 80.57 ^Aa^	567.99 ± 119.62 ^Aa^	562.28 ± 95.89 ^Aa^
	day 5	445.92 ± 36.50 ^Aa^	381.82 ± 67.16 ^Ba^	386.14 ± 48.24 ^Ba^	394.02 ± 51.38 ^Ba^
ACE	day 0	555.40 ± 134.83 ^Aa^	594.84 ± 95.81 ^Aa^	618.98 ± 140.68 ^Aa^	604.52 ± 108.44 ^Aa^
	day 5	472.07 ± 39.62 ^Aa^	401.69 ± 72.49 ^Ba^	409.89 ± 53.43 ^Ba^	415.96 ± 56.92 ^Ba^
Shannon	day 0	5.67 ± 0.63 ^Aa^	5.88 ± 0.41 ^Aa^	5.99 ± 0.48 ^Aa^	5.68 ± 0.30 ^Aa^
	day 5	5.03 ± 0.55 ^Aa^	4.38 ± 0.82 ^Ba^	4.48 ± 0.62 ^Ba^	4.53 ± 0.65 ^Ba^
Simpson	day 0	0.89 ± 0.06 ^Aa^	0.90 ± 0.05 ^Aa^	0.93 ± 0.02 ^Aa^	0.90 ± 0.04 ^Aa^
	day 5	0.83 ± 0.06 ^Aa^	0.76 ± 0.13 ^Ba^	0.78 ± 0.08 ^Ba^	0.79 ± 0.09 ^Ba^

Values are expressed as mean ± standard deviation. Different lowercase letters in the same row indicate significant differences (*p* < 0.05). For the same indicator, different capital letters in the same column indicate significant differences (*p* < 0.05) between day 0 and 5. LA, lactic acid; AA, acetic acid.

**Table 3:** Mean relative abundance (%) of bacteria at the genus level.

Genus		Treatment			
		Control	1% AA	1% LA	0.8% AA + 0.2% LA
*Pseudomonas*	day 0	39.08 ^Aa^	32.77 ^Ab^	32.04 ^Ab^	43.30 ^Aa^
	day 5	38.76 ^Aa^	32.47 ^Ab^	29.85 ^Ab^	39.59 ^Aa^
*Alkanindiges*	day 0	8.13 ^Aa^	12.35 ^Aa^	10.85 ^Aa^	7.42 ^Aa^
	day 5	5.17 ^Aa^	0.67 ^Bb^	0.33 ^Bb^	1.48 ^Bb^
*Massilia*	day 0	7.74 ^Aa^	6.20 ^Aa^	6.98 ^Aa^	6.59 ^Aa^
	day 5	4.93 ^Aa^	0.28 ^Bb^	0.29 ^Bb^	0.14 ^Bb^
*Sphingomonas*	day 0	6.70 ^Aa^	8.32 ^Aa^	4.48 ^Aa^	5.96 ^Aa^
	day 5	0.39 ^Ba^	0.59 ^Ba^	0.38 ^Ba^	0.34 ^Ba^
*Acinetobacter*	day 0	5.15 ^Aa^	3.00 ^Ab^	8.66 ^Aa^	5.98 ^Aa^
	day 5	0.26 ^Ba^	0.76 ^Bb^	0.51 ^Bb^	0.44 ^Bab^
*Escherichia-Shigella*	day 0	0.01 ^Aa^	0.01 ^Aa^	0.00 ^Aa^	UD ^Aa^
	day 5	33.09 ^Ba^	55.34 ^Bb^	58.82 ^Bb^	50.71 ^Bb^

Different lowercase letters in the same row indicate significant differences (*p* < 0.05). For the same bacteria, different capital letters in the same column indicate significant differences (*p* < 0.05) between days 0 and 5. UD, undetected; LA, lactic acid; AA, acetic acid.

## References

[1] Wang J., Tao D., Wang S., Li C., Li Y., Zheng F., Wu Z. (2019). Disinfection of lettuce using organic acids: An ecological analysis using 16S rRNA sequencing. RSC Adv..

[2] Wang J., Wang S., Sun Y., Li C., Li Y., Zhang Q., Wu Z. (2019). Reduction of *Escherichia coli* O157:H7 and naturally present microbes on fresh-cut lettuce using lactic acid and aqueous ozone. RSC Adv..

[3] Ölmez H., Kretzschmar U. (2009). Potential alternative disinfection methods for organic fresh-cut industry for minimizing water consumption and environmental impact. LWT-Food Sci. Technol..

[4] Akbas M.Y., Olmez H. (2007). Inactivation of *Escherichia coli* and *Listeria monocytogenes* on iceberg lettuce by dip wash treatments with organic acids. Lett. Appl. Microbiol..

[5] Huang Y., Chen H. (2011). Effect of organic acids, hydrogen peroxide and mild heat on inactivation of *Escherichia coli* O157:H7 on baby spinach. Food Control.

[6] Samara A., Koutsoumanis K.P. (2009). Effect of treating lettuce surfaces with acidulants on the behaviour of *Listeria monocytogenes* during storage at 5 and 20 °C and subsequent exposure to simulated gastric fluid. Int. J. Food Microbiol..

[7] Yuk H.G., Yoo M.Y., Yoon J.W., Moon K.D., Marshall D.L., Oh D.H. (2006). Effect of combined ozone and organic acid treatment for control of *Escherichia coli* O157:H7 and *Listeria monocytogenes* on lettuce. J. Food Sci..

[8] Singla R., Ganguli A., Ghosh M. (2011). An effective combined treatment using malic acid and ozone inhibits *Shigella* spp. on sprouts. Food Control.

[9] Yuk H.G., Yoo M.Y., Yoon J.W., Marshall D.L., Oh D.H. (2007). Effect of combined ozone and organic acid treatment for control of *Escherichia coli* O157: H7 and *Listeria monocytogenes* on enoki mushroom. Food Control.

[10] Zhang J., Yang H. (2017). Effects of potential organic compatible sanitisers on organic and conventional fresh-cut lettuce (*Lactuca sativa* Var. *Crispa* L). Food Control.

[11] Adams M.R., Hall C.J. (1988). Growth inhibition of food-borne pathogens by lactic and acetic acids and their mixtures. Int. J. Food Sci. Technol..

[12] Helander I.M., Wright A.V., Mattila-Sandholm T.M. (1997). Potential of lactic acid bacteria and novel antimicrobials against Gram-negative bacteria. Trends Food Sci. Technol..

[13] Nout M.J.R., Rombouts F.M. (1992). Fermentative preservation of plant foods. J. Appl. Microbiol..

[14] Anderson M.E., Marshall R.T., Dickson J.S. (1992). Efficacies of acetic, lactic, and two mixed acids in reducing numbers of bacteria on surfaces of lean meat. J. Food Saf..

[15] Singleton V.L., Orthofer R., Lamuela-Raventós R.M. (1999). Analysis of total phenols and other oxidation substrates and antioxidants by means of folin-ciocalteu reagent. Methods Enzymol..

[16] Magoč T., Salzberg S.L. (2011). FLASH: Fast length adjustment of short reads to improve genome assemblies. Bioinformatics.

[17] Caporaso J.G., Kuczynski J., Stombaugh J., Bittinger K., Bushman F.D., Costello E.K., Huttley G.A. (2010). QIIME allows analysis of high-throughput community sequencing data. Nat. Methods.

[18] Chen H., Jiang W. (2014). Application of high-Throughput sequencing in understanding human oral microbiome related with health and disease. Front. Microbiol..

[19] Edgar R.C. (2010). Search and clustering orders of magnitude faster than BLAST. Bioinformatics.

[20] Van Haute S., Uyttendaele M., Sampers I. (2013). Organic acid based sanitizers and free chlorine to improve the microbial quality and shelf-life of sugar snaps. Int. J. Food Microbiol..

[21] Poimenidou S.V., Bikouli V.C., Gardeli C., Mitsi C., Tarantilis P.A., Nychas G.J., Skandamis P.N. (2016). Effect of single or combined chemical and natural antimicrobial interventions on *Escherichia coli* O157:H7, total microbiota and color of packaged spinach and lettuce. Int. J. Food Microbiol..

[22] Martínez-Sánchez A., Allende A., Bennett R.N., Ferreres F., Gil M.I. (2006). Microbial, nutritional and sensory quality of rocket leaves as affected by different sanitizers. Postharvest Biol. Technol..

[23] Lopez-Velasco G., Welbaum G.E., Boyer R.R., Mane S.P., Ponder M.A. (2011). Changes in spinach phylloepiphytic bacteria communities following minimal processing and refrigerator storage described using pyrosequencing of 16S rRNA amplicons. J. Appl. Microbiol..

[24] Shen Y., Nie J., Dong Y., Kuang L., Li Y., Zhang J. (2018). Compositional shifts in the surface fungal communities of apple fruits during cold storage. Postharvest Biol. Technol..

[25] Langsrud S., Sidhu M.S., Heir E., Holck A.L. (2003). Bacterial disinfectant resistance—a challenge for the food industry. Int. Biodeterior. Biodegradation.

[26] Lugtenberg B. (2015). Principles of Plant-Microbe Interactions.

[27] Kamilova F., Kravchenko L.V., Shaposhnikov A.I., Makarova N., Lugtenberg B. (2006). Effects of the tomato pathogen *Fusarium oxysporum* f. sp. radices-Lycopersici and Pseudomonas fluorescens WCS365 on the composition of organic acids and sugars in tomato root exudate. Mol. Plant Microbe Interact..

[28] Kamilova F., Validov S., Azarova T., Mulders I., Lugtenberg B. (2005). Enrichment for enhanced competitive plant root tip colonizers selects for a new class of biocontrol bacteria. Environ. Microbiol..

[29] de Weert S., Vermeiren H., Mulders I.H.M., Kuiper I., Hendrickx N., Bloemberg G.V., Vanderleyden J., De Mot R., Lugtenberg B.J.J. (2002). Flagella-driven chemotaxis towards exudate components is an important trait for tomato root colonization by *Pseudomonas fluorescens*. Mol. Plant Microbe Interact..

[30] de Weert S., Kuiper I., Lagendijk E.L., Lamers G.E.M., Lugtenberg B.J.J. (2004). Role of chemotaxis toward fusaric acid in colonization of hyphae of *Fusarium oxysporum* f. sp. radices-Lycopersici by Pseudomonas fluorescens WCS365. Mol. Plant Microbe Interact..

[31] Cherrington C.A., Hinton M., Mead G.C., Chopra I. (1991). Organic acids: Chemistry, antibacterial activity and practical applications. Adv. Microb. Physiol..

[32] Ricke S.C. (2003). Perspectives on the use of organic acids and short chain fatty acids as antimicrobials. Poult. Sci..

[33] Wang C., Chang T., Yang H., Cui M. (2015). Antibacterial mechanism of lactic acid on physiological and morphological properties of *Salmonella* Enteritidis, *Escherichia coli* and *Listeria monocytogenes*. Food Control.

[34] Critzer F.J., Doyle M.P. (2010). Microbial ecology of foodborne pathogens associated with produce. Curr. Opin. Biotechnol..

[35] Ofek M., Hadar Y., Minz D. (2012). Ecology of root colonizing *Massilla* (Oxalobacteraceae). PLoS ONE.

[36] Erlacher A., Cardinale M., Grosch R., Grube M., Berg G. (2014). The impact of the pathogen *Rhizoctonia solani* and its beneficial counterpart *Bacillus amyloliquefaciens* on the indigenous lettuce microbiome. Front. Microbiol..

[37] Akbas M.Y., Olmez H. (2007). Effectiveness of organic acid, ozonated water and chlorine dippings on microbial reduction and storage quality of fresh-cut iceberg lettuce. J. Sci. Food Agric..

[38] Gil M.I., Selma M.V., Lopez-Galvez F., Allende A. (2009). Fresh-cut product sanitation and was water disinfection: problems and solutions. Int. J. Food Microbiol..

